# A Review and Comparative Analysis of Relevant Approaches of Zero Trust Network Model

**DOI:** 10.3390/s24041328

**Published:** 2024-02-19

**Authors:** Poonam Dhiman, Neha Saini, Yonis Gulzar, Sherzod Turaev, Amandeep Kaur, Khair Ul Nisa, Yasir Hamid

**Affiliations:** 1 Government P.G. College, Idgah Road, Kabari Bazar, Ambala Cantt 133001, India; 2 Government College, Chhachhrauli, Yamuna Nagar 135103, India; profnehasaini@gmail.com; 3 Department of Management Information Systems, College of Business Administration, King Faisal University, Al-Ahsa 31982, Saudi Arabia; 4 Department of Computer Science & Software Engineering, College of Information Technology, United Arab Emirates University, Al Ain 15551, United Arab Emirates; 5 Chitkara University Institute of Engineering and Technology, Chitkara University, Rajpura 140601, India; 6 College of Computer Science and Information Technology, University of Bisha, Al Nakhil, Bisha 67714, Saudi Arabia; 7 Information Security and Engineering Technology, AbuDhabi Polytechnic College, Abu Dhabi 111499, United Arab Emirates

**Keywords:** authentication, zero trust (ZT), cloud computing, network security, blockchain

## Abstract

The Zero Trust safety architecture emerged as an intriguing approach for overcoming the shortcomings of standard network security solutions. This extensive survey study provides a meticulous explanation of the underlying principles of Zero Trust, as well as an assessment of the many strategies and possibilities for effective implementation. The survey begins by examining the role of authentication and access control within Zero Trust Architectures, and subsequently investigates innovative authentication, as well as access control solutions across different scenarios. It more deeply explores traditional techniques for encryption, micro-segmentation, and security automation, emphasizing their importance in achieving a secure Zero Trust environment. Zero Trust Architecture is explained in brief, along with the Taxonomy of Zero Trust Network Features. This review article provides useful insights into the Zero Trust paradigm, its approaches, problems, and future research objectives for scholars, practitioners, and policymakers. This survey contributes to the growth and implementation of secure network architectures in critical infrastructures by developing a deeper knowledge of Zero Trust.

## 1. Introduction

The issue of information security has become increasingly difficult as information technology has continued to advance and find practical applications since the inception of the digital age. The prevalence and intensity of attacks related to cyber-security on networks has risen significantly in recent years. Even medium-sized business data centres can experience more than 100,000 security attacks each day. These attacks can be carried out by a variety of opponents, from solitary hackers to organized cyber-gangs. Their goals may include compromising essential network resources that include software-defined networks or Domain Name Servers, potentially jeopardizing their integrity and functionality [1]. As telecommuting and digital transformation gain traction, traditional company boundaries have diminished, removing digital boundaries entirely. As a result, the growing need for remote access has outpaced the capabilities offered by conventional perimeter safety measures. As a result of this trend, various businesses have been forced to reconsider their approach to network security. As a result, a concept known as Zero Trust Architecture has arisen, concentrating on resource security rather than just on perimeters of network. The security posture of a resource is no longer primarily governed by its network location under this approach [2].

**Figure 1:**
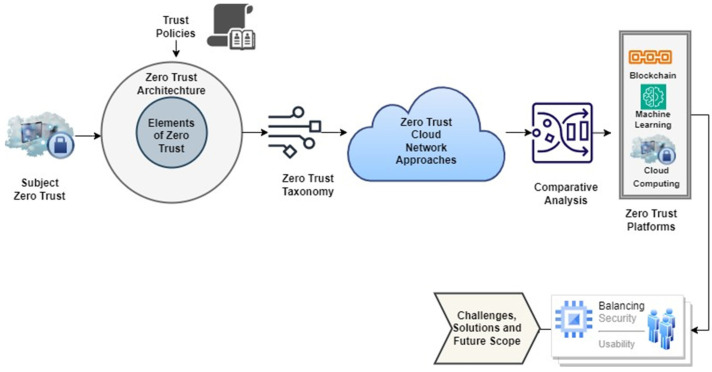
Workflow of study conducted.

Zero Trust Architecture research is now in its early stages, with a primary focus on the framework itself, access control, algorithms of trust evaluation, and identity authentication. These are the primary study domains within the Zero Trust field. Hence, the goal of this research is to provide a complete overview of the current research state in Zero Trust Architecture development, with a focus on four major topics. It covers the primary obstacles encountered in each discipline and investigates potential pathways for future study to successfully address these issues. Figure 1 depicts the work flow of the research article emphasising the interconnections and relationships among different aspects of the conducted analysis.

Our paper contributes significantly in the following ways:

(i) We present a thorough examination of the current state of Zero Trust (ZT) research. We currently focus on the state of research in Zero Trust Architecture framework, access control, evaluation methods, and trust identity authentication. We provide a full knowledge of the general picture of Zero Trust research.
(ii) We compare the most commonly used ZT approaches, provides Zero Trust Architecture (ZTA). The comparison of Conventional vs. ZT Security Model is also performed. We provide a comprehensive comparative assessment of various approaches by exploring their relative advantages and significant obstacles.
(iii) We summarise the key issues faced by these sectors based on the strengths and shortcomings observed in existing Zero Trust network approaches. In addition, we propose critical research directions for the development of zero-trust systems that solve the stated difficulties.

The structure of the paper is organized as: ZTA is introduced in Section 2. Section 3 examines the current state of research on Zero Trust, especially ZT control as well as trust evaluation techniques. In Section 4, we describe the prevailing status of the major systems, compare them, and discuss potential future study options. Finally, we conclude this paper.

### 1.1. Background Study

Even before it was formally referred to as “zero trust,” the notion of ZT was deeply embedded in cyber-security practices. Early breakthroughs in this field included the establishment of the “black core” plan by the Defence Information Systems Agency and the Department of Defence, which attempted to improve enterprise security by changing the focus from perimeter-based protection to safeguarding individual transactions. The Jericho Forum popularized the concept of de-perimeterization in 2004, which entailed minimizing reliance on implicit confidence based purely on network location and recognising the difficulties of depending on single, static defences across a large network segment. De-perimeterization evolved and improved over time, eventually leading to the broader idea known as Zero Trust. John Kindervag coined the phrase “zero trust” while working at Forrester [3]. It soon became a popular phrase for a variety of cyber-security solutions that moved away from relying on implied trust derived from network location and instead prioritized evaluating trust on a transaction-by-transaction basis. Both the corporate sector and higher education institutions have moved away from perimeter-based security and towards ZT security methods. For more than a decade, federal agencies have been encouraged to adopt and implement Zero Trust safety practices. A number of initiatives and policies, including the Federal Information the Trusted Internet Connections, Security Modernization Act, Credential, and Access Management, the Identity based on federalism, and the Continuous Diagnostics and Mitigation programmes, have been established to limit data and resource access to authorized entities [4]. These programmes initially faced restrictions due to the technological limits of information based systems. Safety rules were primarily invariable and imposed at significant “choke points” that organizations could control, with the goal of having greatest impact with the least amount of work. However, as technology has improved, it is now possible to analyse as well as measure access requests in a dynamic and granular manner, based on the idea of “need to access”. This method aids in the reduction of data exposure caused by compromised accounts, network surveillance by attackers, and other security threats. Many researchers have discussed the ZT frame work like Sarkar, Sirshak et al., in which domain-specific issues around cloud computing networks including ZT have been discussed [5]. In this article, challenges associated with cloud platforms only and necessities for transferring to ZTA have been elaborated; unlike in the presented article, we have considered all the applications like cloud computing, blockchain, edge computing, wireless networks, web3 and machine learning that have been incorporated. In this paper, in spite of choosing only specific area like cloud computing, challenges around broader areas have been discussed and reviewed corresponding to various techniques used. The taxonomy of the ZT framework also elaborated in this article.

### 1.2. Elements of Zero Trust

A few elements of ZT are identified and mentioned below:

#### 1.2.1. Implicit Trust Region

It is critical to assume that possible attackers are present within the company network. As a result, all actions involving assets should prioritize security by implementing safeguards such as authentication for all connections and communication encryption.

#### 1.2.2. Bring-Your-Own-Device Policies

The organization employs BYOD policies to accommodate guests, contracted services, and enterprise subjects who use personal devices. When appropriate, this policy allows users to access enterprise resources using non-business-owned equipment.

#### 1.2.3. Security Policy

When moving assets and workloads between enterprise-owned infrastructure and other environments, security must be maintained. Devices such as distant users migrating from business networks to non-enterprise networks, as well as applications shifting across local data centres to non-enterprise cloud scenarios, fall under this category. Enterprise-owned devices may feature artifacts that strengthen authentication and provide a higher level of confidence as compared to non-enterprise devices. It is insufficient to rely exclusively on subject credentials for device authentication in order to access enterprise resources [6].

#### 1.2.4. Assumption of Untrusted Local Network

Remote enterprise topics and assets must assume that their local network connections are untrustworthy. It is critical for remote subjects to anticipate that the local network, which is not owned by the firm, may be hostile. All traffic should be regarded as potentially watched and tampered with by assets. To reduce risks, all connection requests should go through authentication and authorization processes, and communications should be as secure as feasible. This includes assuring the secrecy, integrity, and source authentication of all communications [7].

#### 1.2.5. Less Enterprise-Owned Infrastructure

The resource landscape includes enterprise issues including some cloud based services. In some circumstances, enterprise-owned entities may require accessibility of the local network for providing some network functions and essential connectivity such as DNS resolution.

#### 1.2.6. Multi-Factor Authentication

Implement a system that requires users to produce numerous forms of identification before allowing access. MFV improves security by introducing additional stages of authentication, lowering the chance of un-authorized access due to compromised credentials. This strategy requires attackers to circumvent numerous authentication obstacles [8].

#### 1.2.7. Context-Responsive Access Controls

Few access decisions based on contextual data like device status, user roles, position, and potential risk factors. Access controls can dynamically adjust to provide suitable levels of security by taking into account the present situation and prospective threats or vulnerabilities. This ensures that access is provided or refused based on the context, hence improving overall security [8].

#### 1.2.8. Continuous Monitoring and Validation

Maintain real-time monitoring of user actions, health of gadgets, and application behaviour to identify the possible threats or abnormalities as soon as possible. Validate the effectiveness of security controls, rules, and configurations on a regular basis to verify they are still capable of dealing with developing threats [9].

#### 1.2.9. Data Protection Secure

Implement strong encryption, tokenization, and other data protection mechanisms to safeguard sensitive data during storage, transport, and processing. To prevent unauthorized access to sensitive information, establish strict access controls and deploy comprehensive monitoring procedures.

#### 1.2.10. User and Entity Behaviour Analytics

Use advanced analytics to analyse user and object behaviour, discovering patterns and detecting abnormalities that could signal possible threats or malicious activity. User and entity behaviour analysis help to respond to potential security concerns in organizations proactively [10].

#### 1.2.11. Absence of a Fallacious Perception of Security

The belief that an organization’s employees have undergone initial security screenings is insfficient as a criterion for establishing trust or identifying breaches. It is imperative to hold individuals accountable for their integrity and acknowledge that people may not always adhere to security policies, thereby challenging the validation of trust.

Nevertheless, the Zero Trust model elucidates this notion by implementing an architecture that safeguards against potential insider threats that may go unnoticed. As individuals become acquainted with an organisation or network, they are inclined to recognise the vulnerabilities inside the network boundaries. Insiders, possessing a fundamental understanding of the system design, might pose a greater threat in the event of a security breach due to their awareness of the existing flaws. Therefore, the approach is constructed based on a Zero Trust mindset, necessitating ongoing verification of credentials regardless of the individual involved [11].

### 1.3. Zero Trust Architecture

ZT is a cyber-security concept that prioritizes protection of resources and relies on the grounds that trust should never be presumed but should be constantly reviewed. Identity management, credentials, access management, operational procedures, endpoints, hostile surroundings, and the basic infrastructure are all covered by ZTA. Initially, the emphasis is on limiting resource access to only those with a legitimate need, allowing them only the privileges necessary to complete their jobs (e.g., read, write, delete). Within an organization, a ZTA deployment comprises of various logical connected components that can be implemented as an on-premises service or as a cloud-based service [12]. Figure 1 displays the framework model, which depicts the relationship among these components and their inter linkage. It is vital to remember that this model is an idealized representation of the logical components and their interconnections.

The logical components of a ZTA operate in distinct control and data planes. The Policy Engine is leader of determining resource access based on company policies and external inputs, using a trust algorithm. It collaborates closely with the Policy Administrator (PA) component, which puts the PE’s choices into action. The PA creates or terminates communication pathways between subjects and resources, generates session-specific authentication tokens or credentials, and configures the Policy Enforcement Point (PEP) as needed. Interlinking among subjects and corporate resources are enabled, monitored, and terminated by the Policy Enforcement Point (PEP). It works with the PA to inform requests and receive updates by policy. Although it is an individual logical component, it can be separated into two distinct components. Furthermore, various data sources contribute input and policy. In addition, various data sources supply the PE with input and policy rules for access decision-making [13]. Local and foreign data sources are included. Other important components of a ZT network are:

(i) Continuous diagnostics and mitigation system: gathers information on the present state of enterprise assets and upgrades configuration and software components.
(ii) Industry compliance system: ensures that the corporation is in conformity with essential authoritarian standards, such as healthcare, or financial diligence information based on requirements of security.
(iii) Threat intelligence feed(s): information from internal or external sources that helps the policy engine make access choices.
(iv) Records of network and system activity: combines records of asset operations, network congestion, resource access actions, and other activities to provide actual insights into enterprise data system security posture.
(v) Data access policies: establish the rules and regulations that control access to enterprise resources.
(vi) Enterprise public key infrastructure (PKI) system: generates and tracks certificates of enterprise generated for resources, subjects, services, and requisition [14].
(vii) Identity management (ID) system: ID system generates, maintains, and controls corporate accounts of user and records based on their identity that include subject information, roles, access attributes, and allocated assets.
(viii) Security information and event management system: SIEM gathers and accesses security-based data, allowing policy modification and alerting against potential attacks on organisational assets.

**Figure 2:**
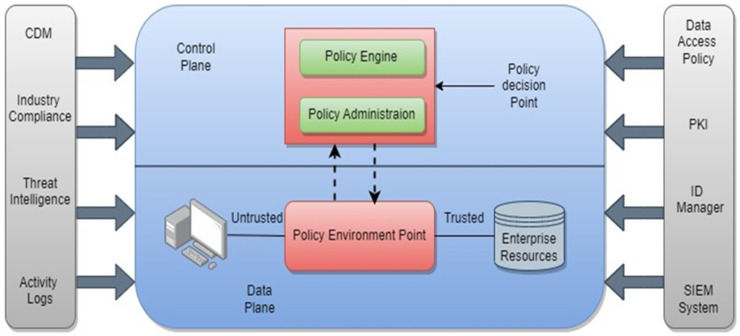
Architecture of Zero Trust networks [4].

Figure 2 depicts the ZT network’s architecture, highlighting the relationships and interactions between different components.

### 1.4. Conventional vs. ZT Security Model

The perimeter (border) security model is the traditional safety paradigm which relies on the foundation of “trust but verify.” It relies on internal users who have passed system or network security functions while being vigilant against external threats. The ZT paradigm, on the other hand, does not presuppose a “trust zone”, and emphasizes verification without inherent trust, even for internal users. In contrast to the perimeter security approach, which lays stress primarily on choking, the ZT model prioritizes complete and ceaseless verification over simply denying access. Table 1 shows a comparison of the traditional security paradigm with the ZT approach.

## 2. Literature Review

Sultana et al. [15] presented a safe medical image allocation platform based on ZT concepts and technology of blockchain. This technology provides the complete security of sensitive medical data. The technology improves information security by utilizing blockchain. However, it is critical to examine the possible complexity and efficiency consequences of combining these technologies, and additional research is needed in this field. Ian and Song [16] suggested a ZT strategy based on the BIBA and BLP models. This method does thorough trust assessments for numerous system components. It emphasizes the importance of confidentiality and integrity and assigns different weights to achieve greater security. It does not, however, address the initial trust value assignment for entities such as users, terminals, environments, and objects, which could lead to human errors. Furthermore, the completeness and logic of the weight assignment list need to be investigated further. Dayna et al. suggested a ZT model specifically intended for cloud data centre networks in a separate study [17] for the creation of trust; their model blends identity management, packet-based authentication, and automated threat response. It controls the model’s eight different network trust levels dynamically.

Traditional network security measures that focus on basics a border between trustworthy and local networks are no longer viable, as cloud apps and IoT networks have become more commonly used. ZT architecture has emerged to meet the need for secure and intelligent access management in the absence of trusted networks or devices. To fulfil the particular security requirements of respective networks, researchers devised and implemented numerous variants of ZTA. Pedro Assuncao proposed a ZT architecture in [18] that eliminates unchanged credentials, uses multifaceted verification, and keeps a proper record of devices and network congestion. In the meantime, ref. [19] proposed a context-based ZT architecture access control system to address security issues in a heterogeneous Moodle application. This framework employs the Zero Trust concept to offer access control for the e-Learning platform Moodle, demonstrating positive webserver performance gains. However, additional tests are required to evaluate the Zero Trust model’s non-functional performance. 

The ZT security framework that featured by continual verification of identity and minimal power distribution, is capable of meeting the safety control needs of various contemporary networked devices. A proposed system for access control and permission relies on the ZT security architecture. Individual identities and confidence from users are derived based on behaviour of users. The system utilizes real-time hierarchy oversight across many settings to effectively accomplish flexible and precise control of access and authentication.

### Taxonomy of Zero Trust Network Features

**Figure 3:**
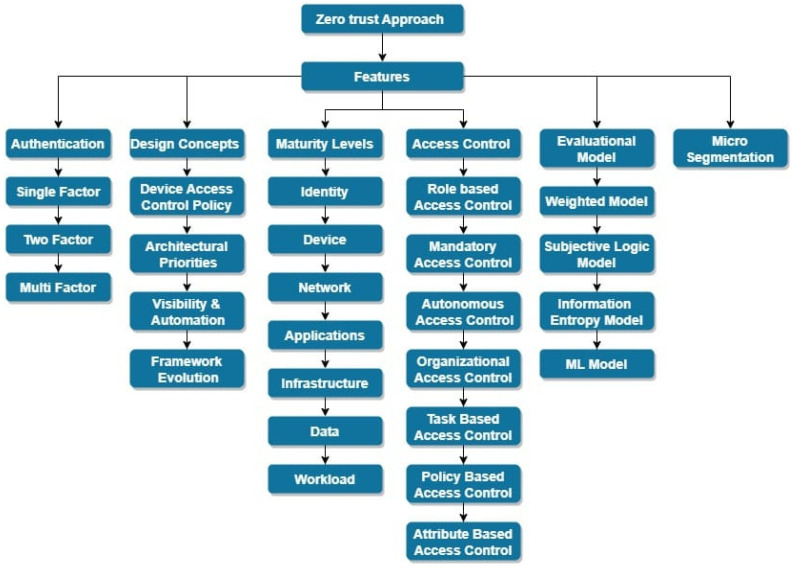
Taxonomy of Zero Trust features.

Figure 3 depicts the way significant features in ZT networks are classified. These features are elaborated as follows:

Authentication: This feature is concerned with validating user credentials in order to distinguish legitimate users from bad actors attempting to obtain unauthorized access. To secure the identification of valid users and to protect devices and data from unauthorized access, robust authentication procedures are required [20].

A ZT network’s design policies include features, such as device access policies, architectural policies, frameworks, and automation. These policies guide the ZT networks’ implementation and operation.

Maturity levels: A ZT network’s maturity is classified into different levels as shown in Figure 2. The conventional level denotes the lack of a ZT implementation, whereas the advanced level denotes the partial implementation of a ZT model. The optimal level denotes complete automation and implementation of the ZT approach.

A continuous evaluation framework is a critical component of a ZT system. The module of trust evaluation analyses and assesses access requests using security data collected by the auxiliary platform, generating trust values. These trust values form the foundation of the authorization mechanism, allowing for dynamic and refined trust assessment.

Micro-segmentation: This method splits the system into small segments having their own security and access control policies set [21]. Micro-segmentation can prevent unapproved usage of crucial information or assets and limit the potential implications of a breach.

Access control: This is an essential prerequisite for ZTA, as it involves the capability to determine the privileges of a subject and subsequently limit access based on those privileges. The primary objective of logical access control is to safeguard resources, including information, components, and programmes by regulating the activities that a subject is permitted to perform on them [22].

In order to successfully execute a designated action on a specified entity, the individual must adhere to the established access control protocols. Specifically, if the prescribed policy requirements are met, permission to interact with the entity is granted.

Overall, these important elements contribute to a ZT network’s efficacy and security by guaranteeing robust authentication, well-defined design principles, incremental maturity levels, fine-grained access control evaluation, and the implementation of micro-segmentation for increased security.

## 3. Comparative Analysis of Zero Trust Network System

Zero Trust is a collection of established and new technologies, rather than a single, rigid design. It is critical to compare various technologies in order to identify which aspects have the greatest or worst fit. Table 2 represents the comparison between different approaches of ZT network based upon some significant parameters. Outdated designs can be changed with more effective ones as opponents progress. The model’s operational requirement takes precedence above its economic efficiency. Furthermore, writers have provided useful insights into how the majority of articles have primarily contributed to the framework design and strategy of ZT networks [23]. Such comparative metrics highlight the common key requirements identified across various cloud network implementations. Beneficiary node cooperation as a distinct group is crucial for network security in an unstable computer network environment. Given the critical necessity of resource security in cloud networks, determining the reliability of requests must rely on past data. Using different rules and standardized methods for the implementing restrictions of access for people in a fragmented network is not a novel approach. Creating a network’s restricted visibility buffer zone serves as the main network’s outer layer [24]. While unauthorized individuals may exploit weaknesses within this zone, attaining access to the network, which is secluded by a strong firewall, becomes hard and evident. Efficient network log keeping is critical, especially in cloud contexts [25]. Log storage in a standardized layout that could be processed by programmed software maximizes the prospective for proactive network security. Network devices must separate and route traffic relied on the association of the service within the organizational composition. Network segmentation improves network security by automating network defences against resource enumeration. Open source software has been a pillar of unrestricted participation in the development of software. Using open-source tools and software, which are freely available and changeable, allows government organizations and enterprises to customize and deploy technologies swiftly [26]. Because of the resource pooling and experience within open source groups, open source tools are normally more secure. Finally, consumer demand drives support for micro services. The packaging of full programmers or software suites improves efficiency and simplifies maintenance. These characteristics were chosen after reviewing the selected studies and reflect the common key criteria observed across numerous ZT network implementations [27].

As the fundamental basis for cloud platforms, ZT ought to encourage the reconsideration and reprioritization of existing end-user technologies. Biometric-based authentication approaches have been widely proposed, taking use of the uniqueness and persistence of human physiological characteristics to authenticate user identity. Kindervag et al. [37], to address the limitations of identity authentication, suggest leveraging devices and applications in android cellphones to accumulate fingerprint data. However, this approach is restricted to certain mobile phone models, resulting in incomplete coverage. To enhance security, it is crucial to move beyond single-factor authentication systems and explore improvements. A critical area that requires attention is the automated classification and detection of attack signature, which has long been identified as a crucial step towards bolstering cyber-security. An experimental testing ground demonstrating the usage of dynamic control plane feedback relied on observes, orient, and decide, Act architecture was provided in their study [38]. The test bed serves as a prototype for a ZT cloud networks. The trial findings given by the author coupled identity managing with threat response automatically and packet-based authentication with management of eight trust levels. Chen and Qiao designed a smart healthcare mechanism for 5G networks in [39], categorizing the system into four dimensions. In the context of ZT and cloud networks, these research initiatives emphasize the need of using biometric authentication, automating security responses, and utilizing machine learning for fine-grained access control. Table 3 represents the techniques used by different studies for implementing the ZT networks, including their advantages and disadvantages.

Because edge computing deployments are dynamic and shared, entities cannot be trusted indefinitely. It is becoming increasingly vital to incorporate ZT policies into (5G/6G) next-generation networks. This review underlines the problems and introduces the notion of intelligent Zero Trust Architecture as a security mechanism in un-trusted 5G/6G communication networks [38].Integrating smart appliances, sensors, and devices with the Internet of Things framework could result in a connected or smart environment [44]. These devices monitor physical environments, send data to a centralized database, and allow for advanced analytics. These IoT devices engage in device-to-device interactions while delivering services to various processes and applications, accessing subsets of device information and data. The receptive characteristics of this data can expose smart infrastructures to a plethora of cyber threats, possibly jeopardizing essential services and people’s lives. Enhanced procedures such as dynamic access control, enhanced network screening, and behavioural anomaly monitoring are required to secure smart settings. The emphasis on network security has altered as networks have switched from conventional in-house servers to remote cloud platforms. The norm has become reactive networks with low accountability and monitoring. Emerging technologies, such as Zero Trust network architecture, have changed the way cloud network security is approached [45]. No entity, regardless of origin or access scope, is implicitly trusted in ZT network access. Instead, the network rewards trustworthy behaviour and forecasts dangers proactively based on user behaviour. Web3, often known as the next-generation web, seeks to build a decentralized and user-controlled online environment [46]. However, it confronts energy consumption, regulatory compliance, interoperability, and scalability concerns. The use of AI technology in ZT network components has garnered little attention in the existing literature. The primary focus of this survey revolves around the utilization of AI technologies to automate and orchestrate components of ZT network architecture. The survey places particular emphasis on the seamless integration of AI-driven automation and orchestration. It is worth noting that edge computing plays a pivotal role in enabling cloud computing and information technology services to be delivered at the network’s edge [47]. The open architecture of cloud computing and network access at the edge, on the other hand, exposes various attack routes [48]. Table 4 represents the comparative analysis of ZT techniques used by different platform.

This section outlines an analysis aimed at examining user perspectives on the usability and security aspects within the context of a zero trust framework. Table 5 balancing security and usability with Zero Trust. As efforts are made to enhance the security of systems, legitimate users may attempt to discover loopholes and devise alternative methods, leading to the compromising of security measures. The issue at hand has been acknowledged by scholars in the field of information privacy and accessibility research. However, progress in addressing this problem has been limited, mostly due to two key factors. Historically, the consideration of security and usability requirements for systems has often been relegated to a secondary position. The strategic plans for creating systems have failed to systematically address and integrate two crucial aspects: security and accessibility difficulties. These two causes have resulted in systems that are often not aligned in terms of privacy and accessibility.

## 4. Challenges, Solutions and Future Scope

The following are some of the challenges that are encountered in the field of Zero Trust:

Technology conjunction: ZT offers the groundwork for re-prioritizing existing end-user technologies and building a simplified approach that minimizes authentication delays while assuring business continuity. ZTA now integrates a number of technologies, including Security Information and event management, trust computation data analytics, file system permissions adjustment via active directory, and multi-factor authentication. These technologies interact with one another. However, there is still tremendous space for advancement and extension in the field of ZT cloud networks, with particular emphasis on the IoT and Block chains, 5G/6G Networks, edge computing and fog computing, and mobile networks.

Security protection capability: While the fundamental aspects of ZT have been developed, ensuring that varied technologies fulfil ZT requirements remains a difficult issue. Access control, identity authentication, and trust evaluation are still in the early stages of development in ZT networks. Future research should concentrate on leveraging these technologies to improve ZTA’s security protection capacity and practicability. Furthermore, deploying newly proposed ZT models to real-world enterprise network systems poses its own set of issues and is an active research topic.

Single-factor authentication: Single-factor authentication is vulnerable to total compromise if the factor, such as a password or biometrics, is stolen. Multifactor authentication, on the other hand, addresses the shortcomings of single-factor authentication while considerably reducing the potential of threat to the network. Even if an invader retrieves the password, acquiring authorization for the second or third factor becomes significantly more difficult. Incomplete authentication information, on the other hand, is insufficient for access. Beyond the initial one-time verification, constant authentication can transform the access information of the attackers. The transition from single-factor to multifactor authentication contributes to ongoing security advancements. ZTA, multifactor and continuous authentication methods are expected to be utilized more in the future. The goal is to minimize resource consumption throughout the authentication procedure while maintaining the ZT system’s security. This is consistent with the future of identity authentication in ZTA.

Complexity and computational cost: Because of the rising complexity and frequency of security assaults against businesses, access control systems must be adjusted according to the need, and assessment of risk must be included into control process. A range of criteria must be considered when making access control decisions, including user and device trust levels, the contextual environment, and the current level of security danger. Furthermore, the access permissions given to devices or people can change with time. In this scenario, risk-based access management is critical. The transition from traditional perimeter-based security models to ZT network security models treats both the workplace intranet as well as internet equally, emphasizing the absence of trust. Reducing authorization and establishing user authorization control that is dynamic are critical issues to address within the present model of ZT access control, which must not be constrained to a single control of access solution.

The continual enhancement of trust assessment comprehensiveness and subjectivity will be a consistent emphasis in future research. Within the scope of ZT theory, attempts are going to be made to increase the efficiency and precision of trust evaluation while considering the specific characteristics of various network environments. Adaptive modification is critical, considering device attributes, place, time, work type, and risk level of security in the environment. Furthermore, the access level allocated to devices or individuals can change over time. The access control standards might be capable of analysing the present degree of trust by utilizing several data sources for decisions making. As a result, access control based on risk should be applied in a range of domains. The underlying trend is a transition away from the old perimeter-based security paradigm and towards the implementation of the Zero Trust security framework, which treats both the workplace intranet and the internet with equal distrust. Within the ZT paradigm, addressing the difficulties of reducing authorization and enabling dynamic authorization control for users is critical. It is vital to stress that the present ZT access control framework be supposed to not be restricted to a single access control strategy, but rather encourage flexibility and adaptation to meet a variety of needs.

## 5. Conclusions

This paper describes in detail the emerging security paradigm known as Zero Trust Architecture. It examines a wide range of implementation strategies and gives an in-depth analysis of zero trust networks essential ideas, including its logical components. Recognizing that no single technology or architecture can fully realize ZT models, the article examines the numerous methodologies and approaches required for its successful implementation. The study emphasizes the necessity of authentication and access control approaches in continuously re-evaluating trust within ongoing connections, emphasizing the value of context, behaviour, and perceived threats. These strategies contribute to the proper implementation of ZTA by taking into account the unique environment of each organization. Furthermore, this article emphasizes the importance of encryption, micro-segmentation, and software-defined perimeters as essential components in building a secure network. The most recent methodologies for applying the indicated security techniques in diverse circumstances are investigated, providing insights into their practical application. This paper also outlines an analysis aimed at examining user perspectives on the usability and security aspects within the context of a zero trust framework. This study serves as a significant resource for scholars, practitioners, and policymakers looking to grasp the essential ideas, approaches, and problems connected with this unique security paradigm by providing a complete review of zero trust models. It establishes the groundwork for future advances in zero trust networks implementation and promotes a more secure and robust approach to network architecture.

## Figures and Tables

**Table 1:** Conventional security model vs. Zero Trust security model.

Comparative Aspects	Conventional Security Model	Zero Trust Security Model
Trust of Stakeholders	They place their faith in internal stakeholders rather than external stakeholders.	Enables traffic to flow only among approved systems, irrespective of stakeholder.
Environment Involves	They have faith in the environment but verify it.	They do not believe anything and double-check everything.
Network Protocol	It uses network protocols for access control.	It employs data-centric control of access.
Communication Channels	Only outward communication channels are encrypted, not internal channels.	Encryption is implemented on the two channels of the whole transmission.
Authentication	Only the initial level of authentication is performed.	Continuous communication phase verification is performed.
Protocols	It is made out of predetermined protocols and security standards.	It comprises customizable security rules and regulations that are evaluated on a regular basis.
Management Approach	It employs a versatile security management approach.	It provides rigorous paradigms as well as a more advanced form of security monitoring.

**Table 2:** Comparison of Zero-Trust cloud networking approaches.

Ref.	Different Levels of Trust	Access Control Standard	Buffer Zone	Logging Procedure	Network Segmentation	Open-Source Software	Micro-Services
[1]		✓		✓	✓		
[28]		✓		✓	✓	✓	
[29]		✓		✓	✓	✓	✓
[30]		✓		✓	✓		
[31]	✓	✓	✓	✓	✓		
[32]		✓		✓	✓	✓	✓
[33]					✓	✓	✓
[34]			✓	✓	✓	✓	
[35]		✓	✓		✓		
[36]		✓	✓	✓			

**Table 3:** Comparative analysis of techniques of Zero Trust networks.

Ref.	Technique Used	Advantage	Disadvantage
[15]	Blockchain	Bringing blockchain and ZT together	Inadequate efficiency
[16]	BLP and BIBA models	Assign various weights on the basis of the need for confidentiality and integrity.	The amount of weight proportion is not appropriate.
[37]	DAN	Real-time data inspection and analysis	As the complexity of networks grows, so does user communication delay.
[38]	Machine learning-based smart association models	API design, maintenance, and monitoring can all be made easier.	In the real world, this is difficult to achieve.
[39]	Artificial intelligence for intelligent detection,	Increase the effectiveness of ZTA modules in interpreting large amounts of data.	Theoretical approach
[40]	Zero Trust, 5G, Attribute-based Access Control	The framework used the ML and DL techniques to handle traffic monitoring, access control, auditing, and load matching, as well as trust evaluation and risk level to flexibly allow execution according to features. Significant organizational adjustments may be required. Adapting to decentralized settings	Relevant to the healthcare environment, and thus concentrated on access to resources instead of requirements for communication.
[41]	Blockchain	Facilitate scalability	Overheads in computation and space
[42]	ABAC, Hyper-Ledger Fabric	Fabric IoT implements ABAC via smart gateways and a hyper-ledger-based architecture.	The most significant drawback for fabric-IoT is scalability, paired with inadequate assistance for applications that use Internet of Things integration.
[43]	NIST NGAC, ABAC access control	The proposed structure involves employing ABAC policies to secure IT devices by defining attributes for Network.	The system employs a collection predetermined policies, and no advance smart dynamic policies can be created at the time of execution to address a advance indeterminate condition.

**Table 4:** Comparison of Zero Trust techniques used by different platforms.

Platform	Applicability	Advantages	Disadvantages	Ref.
IoT	The Internet of Things in Healthcare Securing IoT devices with ZT, trust scores in ZT Campus Networks, supply chain management, context-based access control and and smart farming	Increased security in cloud computing settings for enterprises and prevents unauthorised fingerprinting of protected resources. Increase the efficiency of ZTA components in processing large amounts of data.	Identity-based access control and permission-based policies for iot device security, real-time environment monitoring and behaviour baseline development, i.e., improving policy enforcement points (PDP) decision-making in response to dynamic changes.	[49,50,51]
Cloud Computing	Empowering remote access and collaboration, making software and computing resources more convenient and efficiently available via internet-based solutions, seamless data storage and remote access, transforming communication and catalysing innovations.	Ensuring the privacy of user data in location-based services, enhancing security and trust in identity authentication processes using the client-server model, assessing trustworthiness and improving security in dynamic environments using an analyzing cloud data plane performance under load and its impact on the control plane.	Cloud services with a limited user base, addressing cost and training issues, considerations for cybersecurity in decentralised systems, maintaining user trust and facilitating adoption, mitigating security risks and increasing system confidence.	[45,52]
Blockchain	Increasing knowledge and use of blockchain technology and cryptocurrencies, acceptance of bitcoin, ethereum, and defi platforms in mainstream markets, the nft boom and blockchain’s role in establishing unique ownership for artists, musicians, and content creators are revolutionising digital asset verification.	Cross-chain interoperability is improved, technologies that enhance privacy are provided, scalability and rapid rate of transactions are enabled, and latency is minimised.	Although the use of blockchain technology provides built-in safety benefits that include confidentiality and the use of encryption, it still remains necessary to make sure private data as well as additional confidential data is safeguarded in accordance with relevant privacy regulations. Additionally, computation and space operating costs must be discussed in order to achieve the optimal balance for optimum system performance.	[9,53,54,55]
Edge Computing	Edge computing and its impending implementation in Sixth Generation (6G) mobile networks, as well as Multiple Edge Computing (MEC) in 5G, signal a shift in central computation and application deployment to the network’s edge.	Interoperability across platforms, safe multi-party computation, ultra-low latency time to response, differential privacy, and tremendous bandwidth.	Scalability limitations, as well as difficulties associated with decentralised trust structures, introduce platforms to new cyber threats.	[56,57,58]
Mobile Netwok	Comprehensive enterprise mobile security, tool and technology suite to protect devices, data, and mobile applications, mobile operating system advances: contributing to improved security measures for mobile devices, enterprise mobility management: enabling effective mobile device and data control and security app security vetting and mobile application development: ensuring robust security measures for mobile applications.	Using collaborative decentralised development and open-source software to promote innovation, collaboration, and data privacy protection, improved security in multiple dimensions-protecting users, terminals, and applications, identity security authentication continuous trust assessment, granular authorization mechanisms for improved security.	The legal framework surrounding decentralised innovations continues to evolve, and novel regulations and laws run the danger of stifling innovation and restricting the possible advantages offered by these innovations.	[59,60]
Wireless Network	Configuring restricted usage throughout all services and applications. Configuring auto-VPN for every user, which directs users through the right connection automatically.	It allows for safe virtualization. This will decrease the expense related to compliance as well as additional security audits. This will grow with the organisation plus novel ways for running enterprise, and it will make it simple to manage workloads.	Improving the precision and flexibility of rule creation to improve subjectivity, increasing evaluation efficiency, overcoming technical limitations, identifying solutions to improve overall performance and effectiveness.	[61,62,63,64]
Web3	Getting ready for the Web3 era by improving user and device authentication in decentralized services and resources, securing remote access in Web3, implementing web3 zero-trust access controls.	Boosting protection and safeguarding systems, reducing vulnerabilities and exposure to threats, decentralized identity management, automated security, enhancing authorization and permissions management for better access control, improving protection for cloud-based environments, scalable identity management.	Smooth collaboration and interoperability through seamless integration with decentralized identity systems, adaptability to decentralized application environments.	[65,66,67,68]
ML	Improving diagnoses and treatment plans through medical data processing, supply chain management transformation, tracking products, ensuring authenticity, and enabling transparency and security.	Improving transparency and accountability, automation, using ai-powered algorithms to access data and analyze informed decisions to drive reality based scenario analysis.	Adapting to decentralized ecosystems may necessitate considerable organizational adjustments.	[39,69,70,71]

**Table 5:** Balancing security and usability with Zero Trust.

Users	Model	Number of User Involved	Key Findings	Ref.
Non-expert users	Key-Directory Encryption Systems	52-person	The study diverges from previous research, which mostly focused on tradeoffs related to user-interface design. The findings indicate that individuals possess a certain amount of comprehension regarding high-level security attributes and are capable of making rational trade-offs between these attributes and aspects such as convenience.	[72]
Employee in the HR department of an enterprise	Contextual TA may send an alert system	20 to 30 employee records	When creating and implementing trust algorithms, it is crucial to consider the equilibrium between security, usability, and cost-effectiveness. The repeated request for a subject to undergo reauthentication, in accordance with their mission function and organisational role, might result in usability challenges due to the alignment with historical patterns and established norms.	[4]
Users of IS/IT services n five prominent US organisations across several industries	Developing value-based objectives	35 experts	The goals provide a valuable foundation for evaluating the degree to which systems have achieved security and usability. The objectives additionally serve as a foundation for making decisions on the trade-off between security and usability. Computation and space operating costs must be discussed in order to achieve the optimal balance for optimum system performance.	[73]
Users of financial services industry	Single-factor and two-factor authentication in automated telephone banking	62 telephone banking users	The objective of this study was to investigate user perspectives on the usability and security of single-factor and two-factor authentication techniques within the framework of an existing automated telephone banking service. The findings reveal notable disparities between the two authentication methods. These disparities, along with the preferences expressed by participants during the interview, can provide valuable insights for making informed decisions regarding the implementation of two-factor authentication.	[74]
Internal security teams	User specific security policy through the formal modeling of user behavior	--	One of the advantages of using a Zero Trust approach is the notable augmentation in the level of effort required by intruders to accomplish their goals. However, the implementation of Zero Trust will also result in heightened management complexity for internal security teams. These teams will require a mechanism for gathering data and implementing policy decisions based on analysis. The proposed procedure must be executed across all organisational systems and data, encompassing all access situations.	[75]

## Data Availability

The data presented in this study are available within this article.
